# Evaluation of a continuously active disinfectant for decontamination of portable medical equipment

**DOI:** 10.1017/ice.2021.66

**Published:** 2022-03

**Authors:** Sarah N. Redmond, Jennifer L. Cadnum, Sandra Y. Silva, Basya S. Pearlmutter, Annette L. Jencson, Heba Alhmidi, Brigid M. Wilson, Curtis J. Donskey

**Affiliations:** 1 Case Western Reserve University School of Medicine, Cleveland, Ohio; 2 Research Service, Louis Stokes Cleveland Veterans’ Affairs Medical Center, Cleveland, Ohio; 3 Clinical and Translational Science Program, School of Medicine, Case Western Reserve University, Cleveland, Ohio; 4 Geriatric Research, Education, and Clinical Center, Louis Stokes Cleveland VA Medical Center, Cleveland, Ohio

## Abstract

A single spray application of a continuously active disinfectant on portable equipment resulted in significant reductions in aerobic colony counts over 7 days and in recovery of *Staphylococcus aureus* and enterococci: 3 of 93 cultures (3%) versus 11 of 97 (11%) and 20 of 97 (21%) in quaternary ammonium disinfectant and untreated control groups, respectively.

One limitation of current cleaning and disinfection strategies is that disinfected surfaces rapidly become recontaminated.^1,2^ This limitation has led to interest in the development of technologies that provide continuous decontamination between episodes of manual cleaning.^2^ One promising technology is continuously active quaternary ammonium disinfectants that contain polymer coatings that bind to surfaces resulting in persistent antimicrobial activity.^2^ Rutala et al^3^ reported that a continuously active quaternary ammonium disinfectant demonstrated sustained antimicrobial activity against several pathogens after 24 hours. Others have reported reductions in contamination of surfaces treated with continuously active quaternary ammonium disinfectants, and in 1 study, healthcare-associated infections were reduced.^4–6^

One potential application of continuously active disinfectants is portable medical equipment. Portable devices are often contaminated and have been implicated as a potential vector for transmission.^1^ Current guidelines recommend that medical equipment that comes into contact with intact skin be cleaned and decontaminated after each patient use.^1^ However, cleaning and disinfection of portable equipment is often suboptimal.^1,7^ We hypothesized that application of a continuously active disinfectant would be effective in reducing contamination of medical equipment used in a hospital setting.

## Methods

### Study setting

The Cleveland VA Medical Center is a 215-bed hospital with an affiliated 250-bed long-term care facility (LTCF). Portable medical equipment is stored between use in common areas on each ward. According to hospital policies, equipment should be decontaminated with antimicrobial wipes by the provider after use, but compliance with this policy is not monitored.^7^

### Evaluation of persistent antimicrobial activity of the continuously active disinfectant

Sani-24 germicidal spray is Environmental Protection Agency (EPA)-registered as Firebird F130 (Microban Products, Huntersville, NC) and marketed by Professional Disposables International as Sani-24. The product has a 24-hour residual disinfectant claim.^3^ We used EPA protocol #01-1A to assess persistent activity against methicillin-resistant *Staphylococcus aureus* (MRSA, clinical strain), vancomycin-resistant *Enterococcus faecium* (VRE, VanB-type strain), carbapenem-resistant strains of *Klebsiella pneumoniae* (American Type Culture Collection [ATCC] BAA-1705) and *Enterobacter aerogenes* (clinical strain), *Candida auris* (Centers for Disease Control and Prevention strain 0381), and *Clostridioides difficile* spores (ATCC 43598).^8^ The method requires the use of a standardized abrasion machine to apply multiple wet and dry wiping steps over 24 hours in addition to multiple reinoculations of the pathogen.^3,8^ After 24 hours, the test surface was inoculated with 10^6^ colony-forming units (CFU) of the test pathogen and the log_10_ reduction after 5 minutes was calculated.^3,8^ A standard quaternary ammonium-alcohol disinfectant spray (Cavicide 1 SKU 13-5024) was used for comparison. The test surface was glass slide carriers with 5% fetal calf serum as the organic load. The neutralizer was 1.5% lecithin and 5% Tween 80.

### Evaluation of effectiveness in reducing contamination of portable medical equipment

The evaluation was conducted on wards in the hospital and the LTCF. In total, 114 portable devices were assigned by block randomization to receive no treatment (N = 38), a single spray application of Cavicide quaternary ammonium-alcohol disinfectant (N = 38), or a single spray application of the continuously active disinfectant (N = 38). The devices were sprayed once with adequate product to thoroughly wet the surfaces and allowed to air dry. The equipment included portable vital signs equipment (N = 40), bladder scanners (N = 19), electrocardiogram machines (N = 25), work stations on wheels (N = 17), doppler ultrasounds (N = 5), portable scales (N = 6), an infusion pump (N = 1), and a vein finder (N = 1).

The devices were sampled at baseline and 1, 4, and 7 days after treatment using CultureSwabs (Becton Dickinson, Franklin Lakes, NJ) premoistened with Dey-Engley neutralizer (Remel Products, Lenexa, KS). Prespecified sites were sampled focusing on frequently touched areas; given the variability in devices, the surface area sampled varied. Equipment that could not be located during days 1, 4, or 7 was excluded from analysis. The swabs were processed for total aerobic colony counts, *S. aureus*, and enterococci. A previous study demonstrated that *S. aureus* and enterococci were frequently recovered from equipment.^7^ Use and cleaning of the equipment were not monitored.

### Data analysis

Analysis of variance (ANOVA) with a Tukey honest significance test was used to compare mean aerobic colony counts (CFU) on days 1–7 to baseline levels. A logistic model was used to compare the frequency of contamination with *S. aureus* and/or enterococci on days 1–7. All analyses were performed using R version 3.5.1 statistical software (The R Foundation for Statistical Computing, Vienna, Austria).

## Results

In laboratory testing, the continuously active quaternary ammonium disinfectant resulted in ≥5 log_10_ reduction of the MRSA, VRE, *C. auris*, and carbapenem-resistant *K. pneumoniae* and *E. aerogenes* test strains with 5 minutes of exposure but no reduction in *C. difficile* spores. The standard quaternary ammonium disinfectant resulted in ≤0.5 log_10_ reduction of the test strains.

**Fig. 1.**
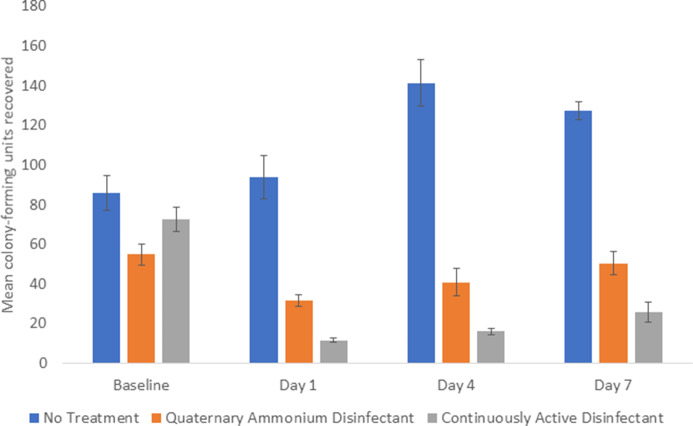
Comparison of total aerobic colony-forming units (CFU) recovered from portable medical equipment at baseline and 1, 4, and 7 days after no treatment (controls) or treatment with a continuously active quaternary ammonium disinfectant or a standard quaternary ammonium disinfectant with no claim for residual antimicrobial activity.

The continuously active disinfectant resulted in sustained significant reductions in aerobic colony counts on equipment in comparison to the baseline level of contamination (*P* < .001) (Fig. 1). The standard quaternary ammonium disinfectant did not result in an overall reduction in aerobic colony counts (*P* = .09). There was no reduction in aerobic colony counts for the untreated control equipment.

**Fig. 2.**
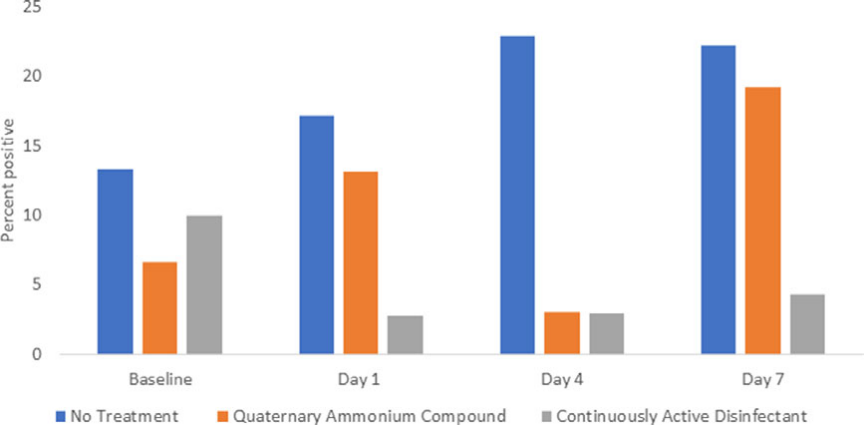
Comparison of *Staphylococcus aureus* and enterococci recovered from portable medical equipment at baseline and 1, 4, and 7 days after no treatment (controls) or treatment with a continuously active quaternary ammonium disinfectant or a standard quaternary ammonium disinfectant with no claim for residual antimicrobial activity.

The percentage of sites positive for *S. aureus* and/or enterococci was significantly reduced on days 1–7 in the continuously active disinfectant group (3 of 93, 3%) versus both the no treatment group (20 of 97, 21%; *P* < .001) and the quaternary ammonium disinfectant group (11 of 97, 11%; *P* = .048) (Fig. 2).

## Discussion

We found that a continuously active disinfectant provided sustained antimicrobial activity against multiple bacterial pathogens and *C. auris*. On hospital and LTCF wards, a single spray application of a continuously active disinfectant on portable medical equipment resulted in sustained reductions in aerobic colony counts over 7 days and reduced recovery of *S. aureus* and enterococci. These results suggest that application of the continuously active disinfectant could be useful to reduce the risk for transmission of pathogens by portable devices.

Our findings are consistent with a recent report from Rutala et al^3^ in which the same continuously active disinfectant provided sustained antimicrobial activity against bacterial pathogens and *C. auris*. Rutala et al^3^ reported only ∼2 log_10_ reduction in carbapenem-resistant *Enterobacter* spp and *K. pneumoniae*, whereas we found >5 log_10_ reductions in these organisms. This difference could potentially be related to differences in test surfaces or other methodologic differences.

Our results suggest that portable medical equipment could be a good place to apply continuously active disinfectant products. Portable devices often become contaminated during medical procedures and patient care activities and are infrequently cleaned.^9,10^ Our data suggest that intermittent application of a continuously active disinfectant could provide sustained antimicrobial activity against bacteria and *Candida* spp.

Our study has several limitations. We did not monitor use and cleaning of the equipment, and we only collected cultures over 7 days. We assessed the impact of the product on *S. aureus* and enterococci. Additional studies are needed to assess additional pathogens. Although the product demonstrated sustained activity despite multiple cycles of dry and wet wiping, it can be removed by chlorine, hydrogen peroxide, and detergents.^3^ Therefore, repeated application would be required in settings where equipment is cleaned with other disinfectants or detergents. Use of the product would not eliminate the need for thorough cleaning prior to application.

In conclusion, application of continuously active quaternary ammonium disinfectants could be useful to reduce contamination of medical equipment. Future studies are needed to determine the impact of these products on colonization and infection with healthcare-associated pathogens.
